# Risk stratification of postoperative pancreatic fistula and other complications following pancreatoduodenectomy. How far are we? A scoping review

**DOI:** 10.1007/s00423-024-03581-9

**Published:** 2025-02-07

**Authors:** Zahraa M. Alhulaili, Rick G. Pleijhuis, Frederik J.H. Hoogwater, Maarten W. Nijkamp, Joost M. Klaase

**Affiliations:** 1 Department of Hepato-Pancreato- Biliary Surgery and Liver Transplantation University Medical Center Groningen, University of Groningen, 30001 9700 RB, Groningen, Netherlands; 2 Department of Internal Medicine University Medical Center Groningen, University of Groningen, Groningen, Netherlands

**Keywords:** Pancreaticoduodenectomy, Risk stratification, POPF, Mortality, Complications, Risk models.

## Abstract

**Purpose:**

Pancreatoduodenectomy (PD) is a challenging procedure which is associated with high morbidity rates. This study was performed to make an overview of risk factors included in risk stratification methods both logistic regression models and models based on artificial intelligence algorithms to predict postoperative pancreatic fistula (POPF) and other complications following PD and to provide insight in the extent to which these tools were validated.

**Methods:**

Five databases were searched to identify relevant studies. Calculators, equations, nomograms, and artificial intelligence models that addressed POPF and other complications were included. Only PD resections were considered eligible. There was no exclusion of the minimally invasive techniques reporting PD resections. All other pancreatic resections were excluded.

**Results:**

90 studies were included. Thirty-five studies were related to POPF, thirty-five studies were related to other complications following PD and twenty studies were related to artificial intelligence predication models after PD. Among the identified risk factors, the most used factors for POPF risk stratification were the main pancreatic duct diameter (MPD) (80%) followed by pancreatic texture (51%), whereas for other complications the most used factors were age (34%) and ASA score (29.4%). Only 26% of the evaluated risk stratification tools for POPF and other complications were externally validated. This percentage was even lower for the risk models using artificial intelligence which was 20%.

**Conclusion:**

The MPD was the most used factor when stratifying the risk of POPF followed by pancreatic texture. Age and ASA score were the most used factors for the stratification of other complications. Insight in clinically relevant risk factors could help surgeons in adapting their surgical strategy and shared decision-making. This study revealed that the focus of research still lies on developing new risk models rather than model validation, hampering clinical implementation of these tools for decision support.

## Introduction

Pancreatoduodenectomy (PD) is the only available cure for patients with pancreatic head and periampullary cancer [1]. The mortality rate following this procedure has decreased significantly over time to below 5% [2–4]. This decrease in mortality rate is due to centralization of care, improvement in peri- and postoperative management as well as the developments in surgical procedures [4–6]. Nevertheless, the overall morbidity rate following PD remains relatively high, between 30 and 50%, with severe complications being reported in approximately 30% of patients [3, 7–9]. Postoperative complications lead to prolonged hospitalization and increased medical costs [10–13]. 

Postoperative pancreatic fistula (POPF) is a serious complication following pancreatoduodenectomy with high prevalence rates between 5 and 30% [10, 14–16]. Clinically relevant postoperative pancreatic fistula (CR-POPF) is defined according to the International Study Group of Pancreatic Surgery (ISGPS) to group B and C [17]. Other common postoperative complications include delayed gastric emptying (DGE), post-pancreatectomy haemorrhage (PPH) and deep and superficial surgical site infection (SSI), all of which could be the sequelae of POPF and lead to prolonged hospitalization and increase in medical costs [10–13, 18, 19]. Other complications that are defined using the ISGPS are postpancreatectomy acute pancreatitis (PPAP), chyle leak, and bile leak [20–22]. According to literature, there are several preoperative factors increasing the risk of POPF and other complications, such as advanced age, male gender, high American Society of Anaesthesiology (ASA) score, malnutrition, high body mass index (BMI), diabetes and cardiovascular disease [6, 23]. Intraoperative factors mentioned to increase the risk of POPF are a narrow pancreatic duct (less than 3 mm), soft pancreatic texture, pancreatic steatosis, absence of pancreatic fibrosis and intraoperative blood loss [2, 6, 13, 23]. Furthermore, several postoperative inflammatory and blood tests are reported to stratify POPF risk, including pancreatic (drain) amylase, white blood cell counts (WBCs), C-reactive protein (CRP), albumin and bilirubin [6, 11, 24, 25]. 

The high prevalence rate of POPF and its severe consequences, such as haemorrhage, inflammation, sepsis, organ failure or even death, makes it crucial to investigate the associated risk factors as well as predicting its risk of occurrence [26]. In this way, perioperative measures can be taken to ensure patient safety and improve postoperative management, both in order to reduce morbidity rates [4]. Over the years, many risk stratification tools were developed to support patient counselling and adapt pancreatic surgery strategy based on estimated individual POPF risk.

The main objective of this scoping review study was to summarize the risk factors used in all published online calculators, equations, nomograms, and artificial intelligence algorithms predicting POPF risk and other complications following PD. In addition, we aimed to provide insight into the various risk factors used and the extent to which risk stratification tools were validated, as deemed mandatory prior to clinical implementation.

## Materials and methods

### Search strategy

**Fig. 1:**
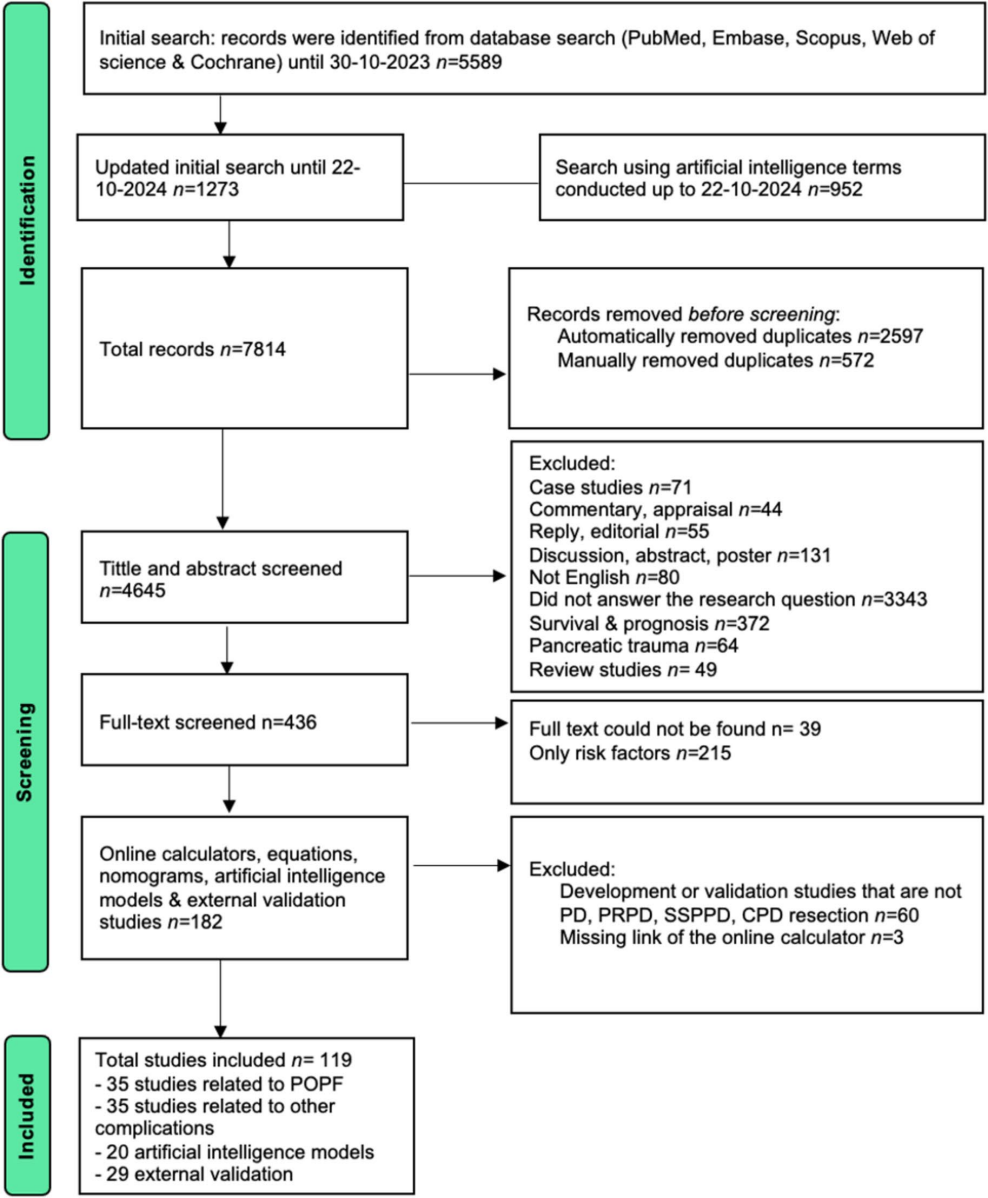
Flow chart of the search strategy **Only risk factors:** studies that only mentioned risk factors for POPF or other complications without developing a risk calculator, equations, or nomogram were excluded

This scoping review study was conducted in consultation with a medical librarian from our institution. An initial search was performed in the following databases: PubMed, Embase, Scopus, Web of Science, and Cochrane to retrieve relevant articles, which was completed by 30th October 2023 as shown in the PRISMA flow diagram Fig. 1 [27] The search terms used that were related to the surgical approach were: pancreaticoduodenectomy, pancreatic surgery, pancreatectomy, Whipple, and pancreatic resection. In addition, the following terms related to risk stratification were used: nomogram, risk calculator, predictive score, online tool, prediction model, risk model, validation, and external validation. Moreover, the following terms related to complications were used: pancreatic fistula, postoperative pancreatic fistula, morbidity, mortality, hemorrhage, bile leakage, delayed gastric emptying, gastroparesis, ISGPS, chyle leakage, failure to rescue, comprehensive complication index, postoperative complications, adverse effects, and postpancreatectomy acute pancreatitis. This initial search was then updated to include studies published up to 22 October 2024 using the same search terms. Figure 1 Artificial intelligence models were searched and included up to 22 October 2024. Figure 1 This search used the same above-mentioned surgical approach and complications terms. However, the following terms related to risk stratification were used: machine learning, deep learning, artificial intelligence, radiomics, decision tree, and random forest. Appendix 1 demonstrates the full search strategy used in the PubMed database. The search was conducted according to the Preferred Reporting Items for Systematic reviews and Meta-Analyses extension for Scoping Reviews (PRISMA-ScR) Appendix 2 [28]. Approval from the ethics committee at our hospital was not applicable due to the nature of this study.

### Inclusion and exclusion criteria

Publications that reported calculators, equations, nomograms, artificial intelligence prediction models that addressed POPF, and other postoperative complications were included in this study. Only pancreatoduodenectomy (PD), pylorus preserving pancreatoduodenectomy (PPPD), pylorus-resecting pancreatoduodenectomy (PRPD), subtotal stomach-preserving pancreatoduodenectomy (SSPPD) and cephalic pancreatoduodenectomy (CPD) were considered applicable in this review. Minimally invasive techniques used in one of the aforementioned resections were also included. All other types of pancreatic resections namely, distal pancreatectomy, total pancreatectomy, and central pancreatectomy were excluded. There was no exclusion of articles based on the publishing date. The search was confined to publications that were written in the English language. The following publications were excluded: congress abstracts, posters, notes, editorials, author replay, appraisal, animal studies, protocols, and case studies. Studies that only mentioned risk factors for POPF or other complications without developing a risk calculator, equations, nomogram, or risk prediction that is based on an artificial intelligence model were excluded.

### Data extraction and reporting

All extracted articles were directly imported into EndNote software version 20.4. Next, duplicates were generated and removed automatically by this software. After that, inclusion and exclusion criteria were applied to the remaining articles. The following data were extracted from the relevant articles: author, country, study design, study period, number of patients, resection type, outcome of interest, definition of complications, risk stratification method, number of risk factors, type of risk factors, involvement of a validation cohort, and external validation. Risk of bias and quality of the included studies were assessed using the PROBAST (Prediction model Risk Of Bias ASsessment Tool) version of 2019, as 3  [29] This tool evaluates risk of bias in multivariable prediction models and to date, it has not been updated to include artificial intelligence prediction models [30]. 

## Results

The initial electronic search in five databases yielded a total of 5589 articles. An update of this initial search resulted in an additional 1273 articles. The search of POPF and other complications using artificial intelligence risk stratification methods yielded 952 articles. The total search resulted in 7814 articles, as illustrated in Fig. 1. After removal of duplicates and assessment of inclusion and exclusion criteria, as shown in Fig. 1, 90 articles were identified as relevant and included in this scoping review. All studies were cohort studies, of which the majority were retrospective in nature, except for eight studies that included a prospective cohort. Most studies were conducted in China (*n* = 33, 36.6%), followed by the United States (*n* = 15, 16.7%). The study periods of the included studies ranged between 1988 and 2024. The included studies were divided into three domains. Studies about independent risk factors and risk stratification of POPF following PD were included in the first domain. In the second domain, the studies regarding independent risk factors and risk stratification for other severe, specific or all complications after PD were included. Studies related to the use of artificial intelligence (AI) algorithms in the development of prediction models following PD were included in the third domain.

### Independent risk factors used in risk stratification of POPF after pancreatoduodenectomy

**Fig. 2:**
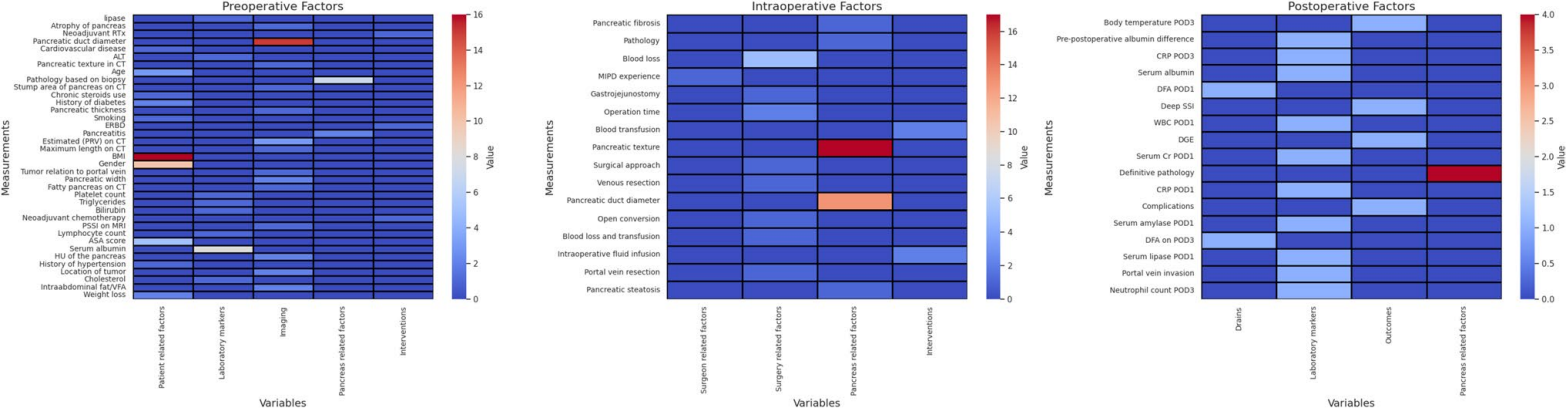
Independent risk factors used in risk stratification of postoperative pancreatic fistula after pancreatoduodenectomy Endoscopic retrograde biliary drainage **(ERBD);** estimated pancreatic remnant volume **(PRV)**; radiotherapy **(RTx)**; drain fluid amylase **(DFA)**; post operative day **(POD)**; minimally invasive pancreaticoduodenectomy **(MIPD);** delayed gastric emptying **(DGE);** surgical site infection **(SSI); **The American Society of Anaesthesiologists **(ASA) **score; alanine transaminase **(ALT)**; creatinine **(Cr)**; White blood cells **(WBCs)**; C-reactive protein **(CRP)**; the pancreatic spleen signal ratio on T1 fat-suppressed MRI sequences **(PSSI)**; Hounsfield units **(HU)**; Body mass index **(BMI)**; visceral fat area **(VFA)**

Thirty-five studies investigating independent risk factors and developing a risk stratification method for pancreatic fistula following PD were identified, as shown in Table 1. Twenty-one studies involved a validation cohort. Studies included solely preoperative factors (*n*= 13), solely intraoperative (*n* = 1) or solely postoperative factors (*n*=1), a combination of pre- and intraoperative factors (*n =* 13), pre- and postoperative factors (*n*= 1), or a combination of all three risk factors (*n =* 6). The most common type of pancreatic resection was PD, which accounted for 80% of the studies, whereas the remaining 20% was distributed between a combination cohort of PD and PPPD (5.7%), CPD (5.7%) and MIPD (8.6%). Five studies developed an online calculator, 14 studies a simple equation and 16 studies a nomogram. The number of risk factors used ranged from 2 to 8, as shown in Table 1. The most applied and investigated risk factor was the main pancreatic duct diameter (*n* = 28, 80%), followed by pancreatic texture (*n* = 18, 51.4%) and Body Mass Index (BMI) (*n* = 16, 45.7%), as demonstrated in Fig. 2. Pathology as a risk factor was used in (*n* = 12, 34.2%) studies, gender in (*n* = 10, 28.6%) studies and preoperative serum albumin in (*n*= 8, 22.9%) studies.

**Table 1:** A summary of online calculators, equations, and nomograms to predict postoperative pancreatic fistula following pancreatoduodenectomy

Study ID	Country	Study design	Study period	Number of patients	Type of resection	Outcome of interest	Outcome definition	Risk stratification method	Number of risk factors	Type of risk factors	Validation cohort included	Externally validated
Callery, M. et al. [31]	USA	PC	January 2002- May 2011	445	PD	POPF	ISGPS 2005 [﻿^32^]	Online calculator	4	Preoperative & intraoperative	Yes	Yes
Mungroop T. et al. [33]	7 European countries	RC	between 2007 and 2017	952	MIPD	POPF	ISGPS 2005	Online calculator	4	Preoperative & intraoperative	Yes	Yes
Mungroop T. et al. [34]	The Netherlands, UK, Italy, USA	PC	Between 2004 and 2016	2850	PD	POPF	ISGPS 2005 & 2016	Online calculator	3	Preoperative & intraoperative	Yes	Yes
Shi, Y. et al. [35]	China	RC	January 2009-November 2019	990	PD	POPF	ISGPS 2016	Online calculator	8	Preoperative	Yes	No
Roberts, K. et al. [6]	UK	RC	February 2007-February 2012	325	PD	POPF	ISGPS 2005	Online calculator	2	Preoperative	Yes	Yes
Chen, J. et al. [36]	China	RC	January 2008- December 2013	921	PD	POPF	ISGPS 2005	Equation	4	Preoperative & intraoperative	No	No
Utsumi, M. et al. [37]	Japan	RC	April 2008-September 2018	108	PD	POPF	ISGPS 2005	Equation	3	Preoperative	No	No
Li, Y. et al. [11]	China	RC	January 2011- December 2016	298	PD and PPPD	POPF	ISGPS 2016	Equation	4	Preoperative & intraoperative	Yes	Yes
Yamamoto, Y. et al. [14]	Japan	RC	January 2004- December 2009	387	PD	POPF	ISGPS 2005	Equation	5	Preoperative	Yes	Yes
Xingjun, G. et al. [38]	China	RC	January 2014- December 2017	609	PD	POPF	ISGPS 2005	Equation	3	Intraoperative	Yes	No
Wellner, U. et al. [39]	Germany	RC	2001–2010	62	PD	POPF	ISGPS 2005	Equation	5	Preoperative	Yes	Yes
Kolbinger, F. et al. [40]	Germany	RC	September 2012- November 2021	195	PD	POPF	ISGPS 2016	Equation	4	Preoperative	No	No
Hirono, S. et al. [41]	Japan, Taiwan	PC	December 2014-May 2017	3022	PD	POPF	ISGPS 2016	Equation	6	Preoperative & intraoperative	No	No
Raza, S. et al. [42]	UK, Italy	RC	January 2009-April 2021	830	PD	POPF	ISGPS 2016	Equation	4	Preoperative & postoperative	No	No
Kantor, O. et al. [43]	USA	RC	2011–2012	1,731	PD	POPF	ISGPS 2005	Equation	5	Preoperative & intraoperative	Yes	Yes
Savin, M. et al. [44]	Romania	RC	2015–2020	78	CPD	POPF	ISGPS 2016	Equation	3	Preoperative	No	No
Mastalier, B et al. [45]	Romania	RC	2010–2021	105	CPD	POPF	ISGPS 2016	Equation	6	Preoperative, intraoperative & postoperative	No	No
Kim S et al. [46]	Korea	RC	January 2008-December 2019	545	PD	POPF	ISGPF 2016	Equation	4	Preoperative	No	No
van Dongen J et al. [47]	The Netherlands	RC	2014–2020	3271	PD	CR-POPF	ISGPS 2016	Equation	5	Preoperative and postoperative	Yes	No
Matsui H. et al. [48]	Japan	RC	January 2010- December 2020	551	PD	POPF	ISGPS 2016	Nomogram	2	Preoperative	Yes	No
Perri, G. et al. [49]	Italy	RC	July 2017-December 2019	566	PD	POPF	ISGPS 2016	Nomogram	2	Preoperative	Yes	No
Choi, M. et al. [50]	South Korea	RC	September 2012- September 2020	429	MIPD	POPF	ISGPS 2016	Nomogram	8	Preoperative & intraoperative	No	No
Lapshyn, H. et al. [51]	Germany	RC	2012–2018	242	PD	POPF	ISGPS 2016	Nomogram	3	Preoperative	Yes	No
Mohamed, A. et al. [52]	USA	RC	2015–2018	5975	PD	POPF	ISGPS 2016	Nomogram	6	Preoperative, intraoperative & postoperative	No	No
Gu, Z. et al. [53]	USA	RC	2014–2017	3609	PD	POPF	ISGPS 2016	Nomogram	6	Preoperative, intraoperative & postoperative	Yes	No
Guo, C. et al. [54]	China	RC	March 2012- October 2017	306	PD	POPF	ISGPS 2016	Nomogram	4	Preoperative, intraoperative & postoperative	Yes	No
Honselmann, K. et al. [10]	Germany	RC	2012–2017	182	PD and PPPD	POPF	ISGPS 2016	Nomogram	5	Preoperative, intraoperative & postoperative	Yes	No
Li, B. et al. [55]	China	RC	December 2018- October 2020	176	PD	POPF	ISGPS 2016	Nomogram	5	Postoperative	No	No
Shen, J. et al. [56]	China	RC	January 2016- May 2020	501	PD	POPF	ISGPS 2016	Nomogram	4	Preoperative, intraoperative & postoperative	Yes	No
Yin, J. et al. [57]	China	RC	January 2012- December 2016	993	PD	POPF	ISGPS 2016	Nomogram	5	Preoperative, intraoperative	Yes	No
You, Y. et al. [58]	Korea	RC	January 2007- December 2016	1846	PD	POPF	ISGPS 2016	Nomogram	6	Preoperative	No	Yes
Zou, J. et al. [59]	China	RC	April 2015- October 2021	205	PD	POPF	ISGPS 2016	Nomogram	3	Preoperative	Yes	No
Zhang, J. et al. [60]	China	RC	August 2012- June 2020	232	PD	POPF	ISGPS 2016	Nomogram	5	Preoperative & intraoperative	No	No
Zhu Y et al. [61]	China	RC	between July 2015 and May 2022	432	MIPD	POPF	ISGPS 2016	Nomogram	6	Preoperative & intraoperative	Yes	Yes
Vo T et al. [62]	Vietnam	RC	between August 2021 and October 2023	183	PD	CR-POPF	ISGPS 2016	Nomogram	4	Preoperative & intraoperative	No	No

Retrospective Cohort (***RC***), Prospective Cohort (***PC***), Pancreaticoduodenectomy (***PD***), Pylorus-resecting Pancreaticoduodenectomy (***PRPD***), Cephalic Pancreatoduodenectomy (***CPD***), Laparoscopic Pancreaticoduodenectomy (***LPD***), Minimally invasive pancreaticoduodenectomy (***MIPD***), Pylorus-preserving Pancreaticoduodenectomy (***PPPD***), Post-operative Pancreatic Fistula (***POPF***)

### Independent risk factors used in risk stratification of other severe, specific or all postoperative complications following pancreatoduodenectomy.

**Fig. 3:**
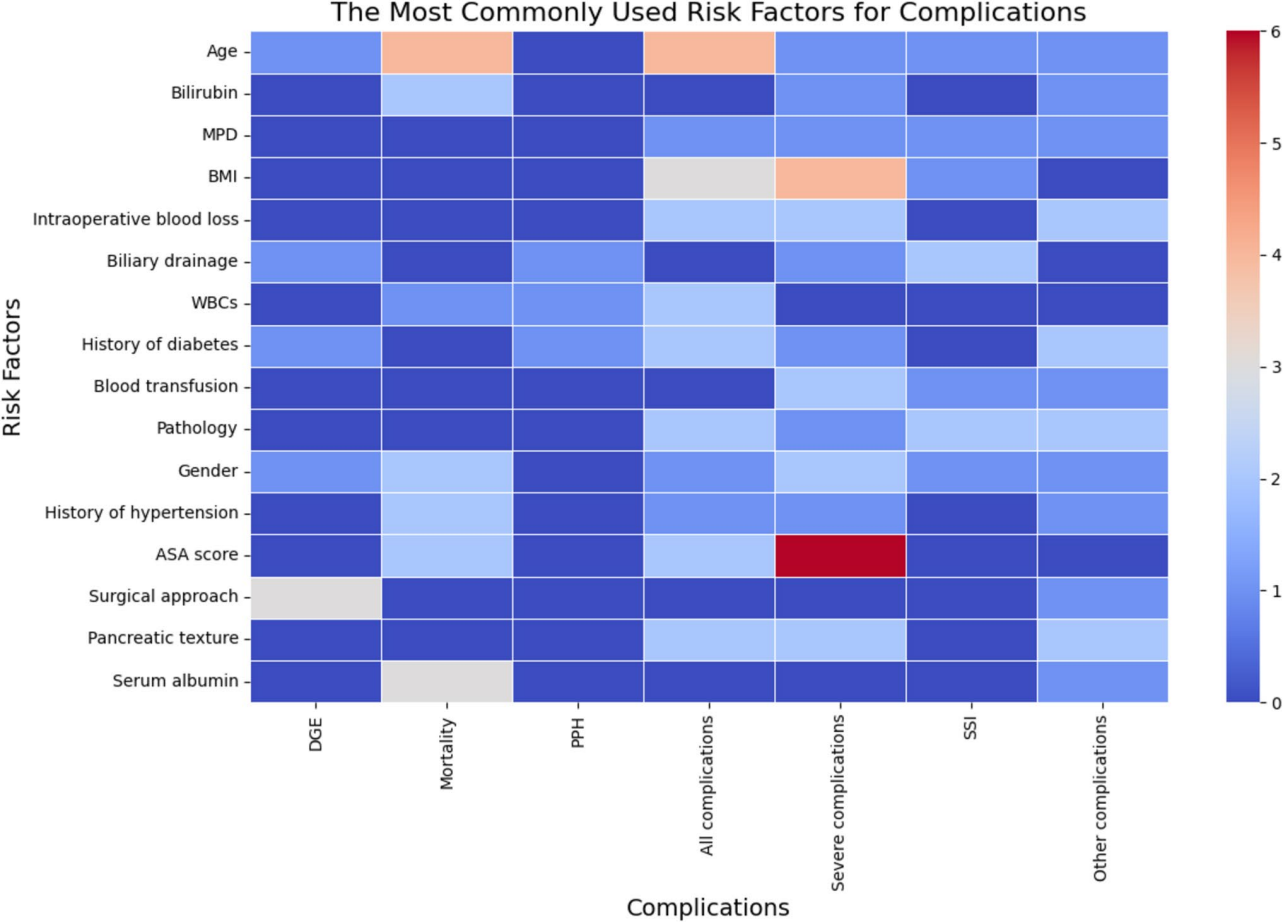
**The most used **risk factors in risk stratification of other complications following pancreatoduodenectomy Body mass index **(BMI)**; The American Society of Anaesthesiologists **(ASA)** score; White blood cells **(WBCs)**; Main pancreatic duct (MPD), Delayed gastric emptying **(DGE), **Postpancreatectomy hemorrhage **(PPH),** Surgical site infection **(SSI)**

Thirty-five studies were obtained from the electronic search regarding postoperative complications after PD as shown in Table 2. In the included studies, severe complications were defined according to Clavien Dindo classification system [102]. The definition of severe complications was in some studies Clavien Dindo IIIa or higher, while in others Clavien Dindo IIIb or higher. Specific complications were defined according to the International Study Group of Pancreatic Surgery (ISGPS), namely POPF, DGE, PPH, PPAP, bile leak and chyle leak [17–22]. The number of variables used in the included studies varied between 2 and 19 risk factors. Nineteen studies involved a validation cohort. Studies used solely preoperative factors (*n =* 13), solely intraoperative factors (*n*= 1), solely postoperative factors (*n*= 6), a combination of pre- and intraoperative factors (*n*= 6), pre- and postoperative factors (*n* = 2), intra- and postoperative factors (*n*= 1), or a combination of all three risk factors (*n*= 6). Most of the studies reported PD as the primary type of resection (74.3%), followed by a combination cohort of PD and PPPD (11.4%). The remaining studies involved PRPD, SSPPD, laparoscopic and robot resection (all together 14.3%). Seven studies focused on risk of all complications, eleven studies on severe complications and/or mortality. DGE was the endpoint in four studies. PPH& SSI were reported separately in three studies. The remaining seven studies were about venous thromboembolism, postpancreatectomy pancreatitis a combination of POPF and DGE, postoperative biliary fistula, postoperative hyperamylasemia and even development of non-alcoholic fatty liver disease (NAFLD) and non-alcoholic steatohepatitis (NASH) and pseudoaneurysm were described. Nine studies developed an online calculator, 15 studies a simple equation and 11 studies a nomogram. The most used risk factors were age (*n* = 12), ASA score (*n* = 10), followed by gender and BMI each in (*n* = 8) studies as illustrated in Fig. 3. Pathology was used as a risk factor in 7 studies, as well as history of diabetes, intraoperative blood loss and pancreatic texture each in 6 studies and pancreatic duct diameter in 4 studies. All risk factors are shown in Fig. 3.

**Table 2:** A summary of online calculators, equations, and nomograms to predict specific, severe or all complications after pancreatoduodenectomy

Study ID	Country	Study design	Study period	Number of patients	Type of resection	Outcome of interest	Outcome definition	Risk stratification method	Number of risk factors	Type of risk factors	Validation cohort included	Externally validated
Al Abbas, A. et al. [63]	USA	RC	2014–2016	9867	PD	All complications	POPF according to Kantor [﻿^43^], Complications own definition	Online calculator	9	Preoperative	No	No
Assifi, M. et al. [64]	USA	RC	January 2000-December 2010	553	PD	Severe complications & mortality	Severe complications Clavien-Dindo ≥ III, POPF ISGPS 2005	Online calculator	3	Intraoperative	No	No
Cai, M et al. [65]	China	RC	January 2010- January 2020	405	PD	All complications	Severe complications Clavien-Dindo ≥ III	Online calculator	4	Preoperative	Yes	No
Choi, M et al. [66]	South Korea	RC	January 2005- December 2014	199	PD and PPPD	Severe complications	According to the ACS NSQIP [67]	Online calculator	19	Preoperative	No	Yes
Sahara, K. et al. [68]	USA	RC	2014–2018	17,683	PD	Mortality	30 days mortality according to ACS-NSQIP [69]	Online calculator	8	Preoperative	Yes	No
Hashimoto Y. et al. [70]	USA	RC	January 1988- July 2008	507	PD	POPF & DGE	DGE 2007, POPF 2005	Online calculator	14	Postoperative	No	Yes
de Castro, S et al. [71]	The Netherlands	RC	January 1993-April 2006	652	PD	All complications & mortality	Morbidity according to Copeland [72], mortality in-hospital death	Online calculator	18	Preoperative & intraoperative	No	Yes
Schroder F et al. [8]	The Netherlands	RC	January 2012- September 2014	110	PD and PPPD	All complications	Severe complications Clavien-Dindo ≥ IIIa	Online calculator	3	Preoperative	No	Yes
Napoli N et al. [73]	Multicenter study (9 countries)	RC	between 2008 and 2020	1184	RPD	Severe complications	Severe complications Clavien-Dindo ≥ III	Online calculator	6	Preoperative	Yes	Yes
Braga, M et al. [74]	Italy	PC	January 2002-August 2010	700	PD	Severe complications	Clavien-Dindo III-IV	Equation	4	Preoperative & intraoperative	Yes	Yes
Chandra, A. et al. [75]	Indonesia	RC	January 2012- October 2022	75	PD	Mortality	Not reported	Equation	4	Preoperative	No	No
Chen, L et al. [76]	China	RC	January 2017-February 2019	159	PD	All complications	Clavien-Dindo ≥ II	Equation	5	Preoperative & intraoperative	No	No
Chen, L et al. [77]	China	RC	2010–2017	183	PD	All complications	Own definition	Equation	4	Postoperative	Yes	No
Palumbo, D. et al. [78]	Italy	RC	January 2013- May 2019	166	PD	PPH	ISGPF 2007	Equation	3	Postoperative	No	No
Zong, K. et al. [79]	China	RC	2018–2022	244	PD	SSI	According to Centres for Disease Control and Prevention [80]	Equation	6	Preoperative	Yes	No
Hu, B. [81]	China	RC	January 2015- March 2019	236	PD	SSI	According to the National Nosocomial Infections Surveillance system [82]	Equation	5	Preoperative, intraoperative & postoperative	Yes	No
Venkat, R et al. [83]	USA	PC	January 1998-June 2009	1976	PD and PPPD	Mortality	30–90 days mortality all causes of postoperative mortality within 30–90 days	Equation	6	Preoperative	Yes	No
Gleeson, E et al. [84]	USA	RC	2005–2012	14,993	PD	Mortality	Postoperative mortality within 30 days	Equation	8	Preoperative	Yes	No
Werba, G. et al. [85]	USA	RC	2014–2018	15,154	PD	DGE	Grade B or C DGE according to ISGPF 2007	Equation	9	Preoperative & intraoperative	No	No
Wiltberger, G et al. [86]	Germany	RC	January 1993- December 2014	405	PD and PPPD	Severe complications	Severe complications Clavien-Dindo ≥ IIIb, POPF IISGPS 2005	Equation	4	Preoperative	Yes	No
Kato, H et al. [87]	Japan	RC	April 2005- October 2008	54	PD, PPPD and SSPPD	NAFLD& NASH	CT Hounsfield units (HU) of less than 40	Equation	5	Preoperative, intraoperative & postoperative	No	No
Morita Y et al. [88]	Japan	RC	Between 2010 and 2020,	120	PD	Pseudoaneurysm	Enlarged arterial diameter with bleeding, irregular arterial wall evaluated by the radiologist	Equation	2	Postoperative	No	No
Birgin E et al. [89]	Germany	RC	January 2009-May 2023	293	PD	PPH	ISGPS 2007 definition of PPH	Equation	4	Postoperative	Yes	No
Addeo P et al. [90]	French	RC	January 2010- April 2018	363	PD	POPF, PPH, DGE, Severe complications	ISGPS systems & Clavien-Dindo ≥ IIIA	Equation	3	Preoperative	No	No
Cai, X et al. [91]	China	PC	December 2009- December 2018	308	PPPD and PrPD	DGE	Grade B or C DGE according to ISGPF 2007	Nomogram	4	Preoperative, intraoperative & postoperative	Yes	No
Hipp, J. et al. [92]	Germany	RC	August 2001-October 2018	956	PD, PPPD, Laparoscopic PPPD	Severe complications	Clavien-Dindo Grades III–V	Nomogram	8	Preoperative, intraoperative & postoperative	Yes	No
Huang, H et al. [93]	China	RC	January 2014 and July 2019	249	PD	All complications	Clavien-Dindo ≥ II, POPF, PPH, DGE according to the ISGPF systems	Nomogram	5	Preoperative & intraoperative	No	No
Shen, Z et al. [94]	China	RC	January 2012- December 2019	835	PD	Severe complications	Clavien-Dindo grading ≥ III	Nomogram	9	Preoperative & intraoperative	No	No
Li, D et al. [95]	China	RC	January 2014- December 2020	409	LPD	PPH	Own definition urgent bleeding from the abdomen or the alimentary tract that needs urgent surgery or embolization	Nomogram	15	Preoperative	No	No
Zhu, L et al. [96]	China	RC	February 2018- October 2021	360	PD	SSI	Temperature of ≥ 38 ◦C, positive drain culture within 3 days after surgery	Nomogram	4	Preoperative & postoperative	Yes	No
Yin, Z et al. [97]	China	RC	January 2018-March 2022	352	PD	VTE	VTE in ultrasound or radiology	Nomogram	13	Preoperative, intraoperative & postoperative	Yes	No
Li, T et al. [98]	China	RC	January 2019- December 2021	422	PD	DGE	Grade B or C DGE according to ISGPF 2007	Nomogram	7	Preoperative, intraoperative & postoperative	Yes	No
Perri G et al. [99]	Italy	RC	January 2018–June 2021	761	PD	POH, POPF, PPAP	ISGPS classification systems	Nomogram	HIST 2, FRS 4	HIST intraoperative, FRS preoperative & intraoperative	Yes	No
Ou Z et al. [100]	China	RC	March 2014 to March 2024	196	PD	POBF	According to Koch M et al. [22]	Nomogram	6	Preoperative & postoperative	Yes	No
Gu Z et al. 101]	USA & China	RC	D 2014–2017 & EV 2014–2019	D 1251 ACS- NSQIP & EV 934	PD	DGE	ISGPS of DGE 2007	Nomogram	4	Postoperative	Yes	Yes

Retrospective Cohort (***RC***), Prospective Cohort (***PC***), Pancreaticoduodenectomy (***PD***), Pylorus-resecting Pancreaticoduodenectomy (***PRPD***), Laparoscopic Pancreaticoduodenectomy (***LPD***), subtotal stomach-preserving pancreatoduodenectomy (***SSPPD***), Minimally invasive pancreaticoduodenectomy (***MIPD***), Pylorus-preserving Pancreaticoduodenectomy (***PPPD***), Post-operative Pancreatic Fistula (***POPF***),Delayed Gastric Emptying (***DGE***), postpancreatectomy hemorrhage (***PPH***), Surgical Site Infection (***SSI***), Venous Thrombo-embolism (***VTE***), nonalcoholic fatty liver disease (***NAFLD***), nonalcoholic steatohepatitis(***NASH***)histopathologic characteristics (***HIST***),Fistula Risk Score(***FRS***),postoperative hyperamylasemia (***POH***), postpancreatectomy acute pancreatitis (***PPAP***), postoperative biliary fistula (***POBF***), Robotic pancreatoduodenectomy (***RPD***)

### Prediction of POPF and other complications after pancreatoduodenectomy using artificial intelligence algorithms

**Fig. 4:**
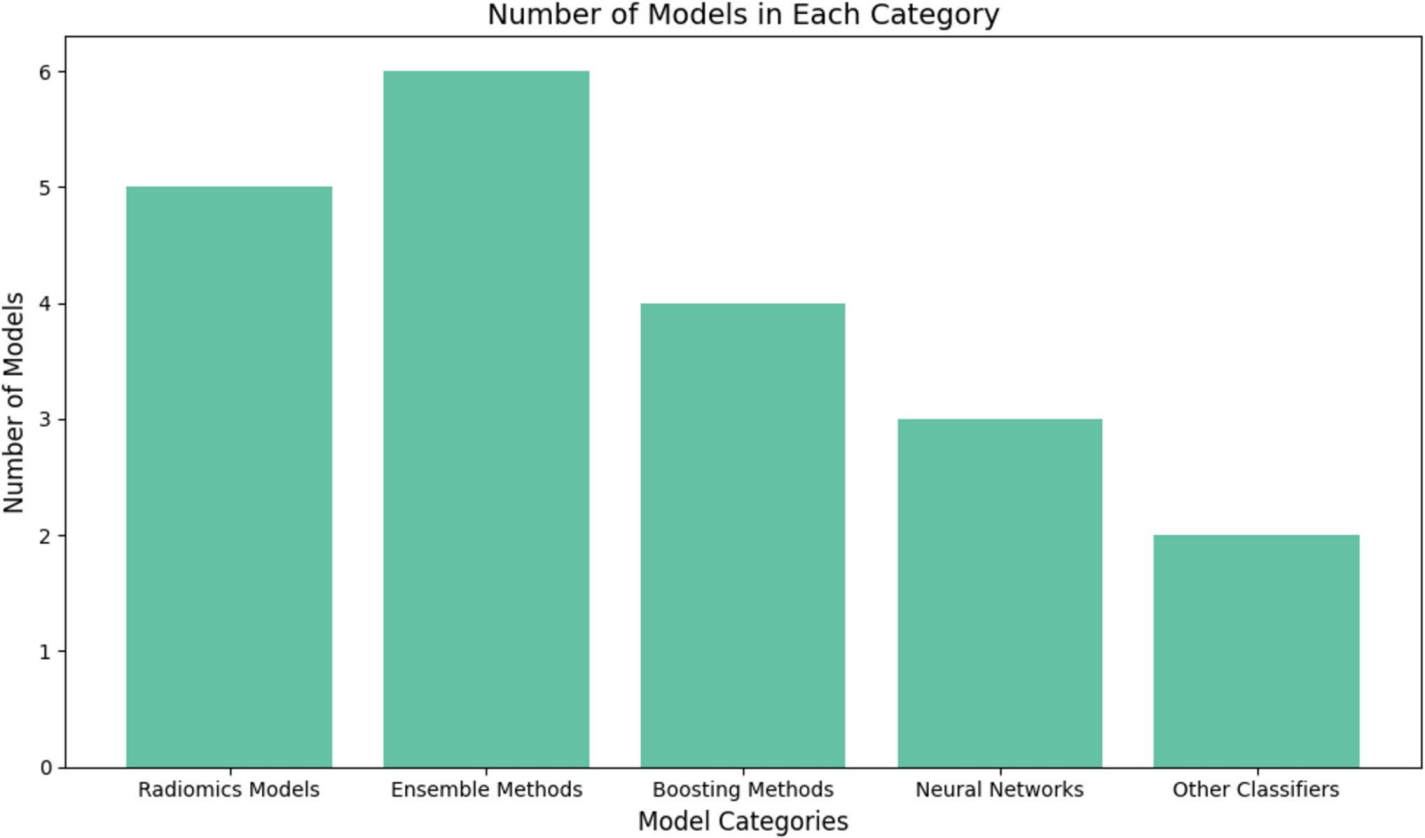
Artificial intelligence methods that were used to develop risk models to predict postoperative pancreatic fistula and other complications following pancreatoduodenectomy

In total, there were twenty studies found that used artificial intelligence in the development of prediction models of which six used ensemble model methods (combination of decision tree and random forest (*n*= 1), ensemble model of machine learning (ML) and deep learning (DL) (*n*= 1), random forest (*n*= 3) and extra tree classifier (*n*= 1)), five studies used radiomics methods (radiomic and clinical signature (*n*= 2), radiomic and clinical features (*n*= 1), radiomics based formula “RAD-score” (*n*= 2)), four studies used boosting methods (gradient boosted tree (GBT) (*n *= 1), XGboost (*n *= 2), CatBoost (*n*= 1)), three models used convolutional neural network methods and the last two studies used other methods namely Gaussian Naive Bayes (GNB) (*n =* 1) and REPTree classifier (*n*= 1) Fig. 4. The most studies used only preoperative risk factors (*n =* 13, 65%). Open PD was performed the most (*n*= 17, 85%), of the remaining three studies, one study had a combination resection of open PD and robot-assisted PD, one study a combination of open PD and minimally invasive approaches, and the last study was about a combination of PD, PPPD and SSPPD Table 3.

**Table 3:** An overview of prediction models based on artificial intelligence algorithms to predict POPF and other complications after PD

Study ID	Country	Study design	Study period	Number of patients	Type of resection	Outcome of interest and definition	Risk stratification method and Model type	Number of variables	Type of variables	Validation cohort included	AUC
Ganjouei A et al. [103]	USA	PC	from 2014 to 2019 ACS-NSQIP database	8666	PD	CR- POPF & severe complications	Online calculator XGBoost	24	Preoperative	Testing set	0.78
Bhasker N et al. [104]	Germany	RC	Between 2011 and 2019	108	PD	CR-POPF according to ISGPF	Radiomic and clinical signature	5	Preoperative	Testing set	0.76
Capretti G. et al. [105]	Italy	RC	between 2011 and 2019	100	PD	CR-POPF according to ISGPF & complications Clavien-Dindo ≥ III	Random forest	10	Preoperative	Cross validation	0.74
Dai Y et al. [106]	China	RC	January 2012 to January 2021	269	PD	POPF according to ISGPF	GNB model	6	Preoperative and postoperative	Testing set	0.82
Fujii T et al. [107]	Japan	RC	Between January, 2012 and December, 2020	186	PD	NAFLD	Clinicoradiomic model	8	Preoperative	Testing set	0.80
Han I et al. [16]	Korea	RC	from 2007 to 2016	1769	PD	POPF according to ISGPF	Online calculator Neural network	16	Preoperative and intraoperative	No internal validation	0.74
Ikuta S et al. [108]	Japan	RC	2008–2023	284	PD, PPPD, SSPPD	PPH according to ISGPF	Extra trees classifier	8	Preoperative and postoperative	bootstrap validation	0.98
Ingwersen E et al. [109]	The Netherlands and Italy	RC	January 2013–December 2018	359	PD	CR-POPF according to ISGPF	Online calculator Random forest	3 features in the RAD-FRS	Preoperative	External validation	Development 0.90 & external validation 0.81
Kambakamba P et al. [110]	Switzerland	RC	2008–2018	110	PD	POPF according to ISGPF	REPTree classifier	a total 419 ML configurations	Intraoperative	Cross validation	0.95
Lee W et al. [111]	Korea	RC	between 2016 and 2018	1333	PD	POPF and CR-POPF according to ISGPF	Ensemble model of 3 ML models & two DL models	6	Preoperative	Testing set	0.68
Long Z et al. [112]	China	RC	between January 2012 and August 2021	618	PD	POPF according to ISGPF	Random forest classifier (RFC)	29	Preoperative and intraoperative	Testing set	0.89
Mu W et al. [113]	China	RC	between 2006 and 2019	513	PD	CR-POPF according to ISGPF	Deep learning score using a convolutional neural network	4	Preoperative	External validation	Development 0.85 validation 0.81
Shen Z et al. [114]	China	RC	2010–2021	2421	Open PD & robot-assisted PD	CR-POPF according to ISGPF	CatBoost algorithm	20	Preoperative, intraoperative & postoperative	Cross-validation	0.81
Skawran S et al. [115]	Switzerland	RC	2008 and 2018	62	PD	POPF and CR-POPF according to ISGPS	Gradient-boosted tree (GBT)	5 radiomic features	Preoperative	Testing set	0.90
Verma A et al. [116]	USA	PC	2014 to 2018 from ACS NSQIP, institution 2013 to 2021	12,281 & 445 respectively	Open PD & MIPD	CR-POPF according to ISGPS	XGBoost	24	Preoperative	External validation	0.79
Yoo J et al. [117]	Korea	RC	2013–2017	257	PD	POPF and Survival	Convolutional neural network	3 predictors from CT scan	Preoperative	No validation	0.79
Zhang W et al. [118]	China	RC	Not reported	117	PD	POPF according to ISGPS	Radiomics-based formula (RAD-score)	11	Preoperative	Testing set	0.76
Zheng J et al. [119]	China	RC	2013–2021	257	PD	POPF according to ISGPS	Decision tree & Random forest	10	Preoperative and postoperative	No validation	Decision tree 0.74 & random forest 0.97
Wu H et al. [120]	China	RC	between January 2015 and November 2021	139	PD	CR-POPF according to ISGPS	Radiomic RAD score	3	Preoperative	Bootstrap resamples	0.83
Lin Z et al. [﻿^121^]	China	RC	between April 2013 and December 2019	250	PD	CR-POPF according to ISGPS	Combined model (radiomics features, demographic, radiological information)	18	Preoperative	Testing set	0.86

Retrospective cohort (***RC***), Prospective cohort (***PC***)Machine learning (***ML***), Deep learning (***DL***), Pancreatoduodenectomy (***PD***), Pylorus Preserving Pancreatoduodenectomy, (***PPPD***), Subtotal stomach-preserving pancreatoduodenectomy (***SSPPD***), Minimally invasive pancreatoduodenectomy (***MIPD***),clinically relevant pancreatic fistula (***CR-POPF***), International Study Group of Pancreatic Surgery (***ISGPS***), Gaussian Naive Bayes (***GNB***),Nonalcoholic fatty liver disease (***NAFLD***),Post-pancreatectomy hemorrhage (***PPH***)

### External validation of the included risk models

Of the included 70 studies related to POPF and other complications after PD, only 18 studies (26%) were externally validated Table 4, whereas of the 20 studies regarding the artificial intelligence models only 3 studies were externally validated in the same development studies and one model was externally validated in a separated study. This shows that only 4 (20%) artificial intelligence models were validated. The most extensively validated scores were the original fistula risk score (o-FRS; 19 validations), alternative fistula risk score (a-FRS; 13 validations) and the updated alternative fistula risk score (ua-FRS; 10 validations). The mean area under the curve (AUC) of the o-FRS was 0.71 SD ± 0.10, a-FRS 0.69 SD ± 0.05 and ua-FRS 0.70 SD ± 0.03. Two studies reported model calibration (expected vs. observed values) instead of the AUC. The Birmingham tool was externally validated in 6 studies, whereas the Tokyo calculator was validated in 3 studies. The Braga study achieved the best AUC among all studies, with an AUC of 0.87 SD ± 0.11 [74].

**Table 4:** Risk models that have been externally validated

Study ID of the original study	Name of scoring system	Outcome of interest	Internal validation	Study ID of the validation study	AUC	95% CI	Mean AUC (SD)
Callery, M. et al. [31]	O-FRS (2013)	POPF	Yes	Blunck CK et al. [122] Miller BC et al. [123] Niu C et al. [124] Grendar J et al. [125] Gupta V et al. [126] Pande R et al. [127] Kang JS et al. [128] Lao M et al. [129] Lee B et al. [130] Ryu Y et al. [131] Shinde RS et al. [132] Shubert CR et al. [133] DI Martino M et al. [134] Mungroop T. et al. [34] Hayashi H et al. [135] You J et al. [136] Verdeyen N et al. [137] Schouten T et al. [138] Ramírez-Giraldo C et al. [139]	0.62 0.72 0.61 0.72 0.80 0.94 0.61 0.70 0.67 0.63 0.65 0.90 - 0.70 0.69 0.69 0.86 0.65 0.56	- - 0.57–0.64 - 0.71–0.87 0.68–0.73 - 1.56–10.54 0.62–0.72 0.59–0.67 - - - 0.66–0.74 0.58–0.80 0.61–0.75 0.89–0.97 0.63–0.66 0.35–0.77	0.71 ± 0.10
Han, I. et al. [16]	-	POPF	No	Yoon SJ et al. [140]	0.67	0.64–0.71	0.67
Mungroop T. et al. [33]	ua-FRS (2021)	POPF	Yes	Blunck CK et al. [122] Niu C et al. [124] Pande R et al. [127] Lee B et al. [130] Shinde RS et al. [132] Hayashi H et al. [135] You J et al. [136] Verdeyen N et al. [137] Schouten T et al. [138] Ramírez-Giraldo C et al. [139]	0.65 0.72 0.75 0.68 0.70 0.67 0.71 0.73 0.70 0.72	- 0.68–0.76 0.67–0.77 0.63–0.74 - 0.56–0.77 0.64–0.77 0.74–0.87 0.69–0.72 0.58–0.86	0.70 ± 0.03
Mungroop T. et al. [34]	a-FRS (2019)	POPF	Yes	Blunck CK et al. [122] Niu C et al. [124] Pande R et al. [127] Kang JS et al. [128] Lao M et al. [129] Lee B et al. [130] Ryu Y et al. [131] Shinde RS et al. [132] Hayashi H et al. [134] You J et al. [136] Verdeyen N et al. [137] Schouten T et al. [138] Ramírez-Giraldo C et al. [139]	0.60 0.73 0.75 0.63 0.74 0.69 0.62 0.69 0.69 0.70 0.75 0.70 0.73	- 0.69–0.79 0.67–0.73 - 2.15–12.38 0.64–0.74 0.59–0.66 - 0.58–0.79 0.63–0.76 0.72–0.86 0.68–0.71 0.57–0.88	0.69 ± 0.05
Li, Y. et al. [11]	SUN-FRS	POPF	Yes	Blunck CK et al. [121]	0.69	-	0.69
Yamamoto, Y. et al. [14]	Tokyo (2011)	POPF	Yes	Pande R et al. [127] DI Martino M et al. [134] Ramírez-Giraldo C et al. [139]	0.81 - 0.70	0.42–0.80 - 0.50–0.90	0.75 ± 0.08
Wellner, U. et al. [39]	-	POPF	Yes	DI Martino M et al. [134]	-	-	-
Kantor, O. et al. [43]	m-FRS (2017)	POPF	Yes	Pande R et al. [127] Schouten T et al. [138] Ramírez-Giraldo C et al. [139]	0.70 0.65 0.56	0.61–0.78 0.63–0.66 0.34–0.78	0.64 ± 0.07
Roberts, K. et al. [6]	Birmingham (2014)	POPF	Yes	Pande R et al. [127] Kang JS et al. [128] DI Martino M et al. [134] Verdeyen N et al. [137] Schouten T et al. [138] Ramírez-Giraldo C et al. [139]	0.83 0.64 - 0.61 0.66 0.81	0.62–0.77 - - 0.66–0.81 0.65–0.68 0.67–0.94	0.71 ± 0.10
You, Y. et al. [58]	-	POPF	No	Yoon SJ et al. [140]	0.68	0.63–0.70	0.68
Perri, G. et al. [49]	-	POPF	Yes	Verdeyen N et al. [137] Schouten T et al. [138]	0.69 0.63	0.62–0.78 0.62–0.65	0.66 ± 0.04
Chen, J. et al. [36]	-	POPF	No	Schouten T et al. [138]	0.64	0.63–0.65	0.64
Choi, M et al. [66]	ACS calculator	Severe complications	No	Jiang HY et al. [141]	-	-	-
Hashimoto Y. et al. [70]	-	POPF & DGE	No	Roberts KJ et al. [142]	0.76	0.68– 0.84	0.76
de Castro, S et al. [71]	P-POSSUM	All complications & mortality	No	Karamolegkou AP et al. [143] Pratt W et al. [144] Karabulut A et al. [145]	0.72 - 0.67	0.57–088 - 0.61–0.73	0.69 ± 0.04
Schroder F et al. [8]	-	All complications	No	Alhulaili ZM et al. [146]	0.67	0.60–0.73	0.67
Braga, M et al. [74]	-	Severe complications	Yes	Joliat GR et al. [147] Sah D et al. [148]	0.95 0.80	- 0.54–1.00	0.87 ± 0.11
Gleeson, E et al. [84]	WHipple-ABACUS	Mortality	Yes	Sah D et al. [148] Lalisang A et al. [﻿^149^]	0.89 0.62	0.78–1.00 0.47–0.79	0.75 ± 0.19

Original fistula risk score (***O-FRS***), Postoperative pancreatic fistula (***POPF***), Updated alternative fistula risk score (***ua-FRS***), Alternative fistula risk score ***(a-FRS)***, modified fistula risk score ***(m-FRS)***, American college of surgeons (***ACS***)calculator, Delayed gastric emptying ***(DGE)***, Area under the curve (***AUC***) Standard deviation(***SD***),Confidence interval(***CI***), Operative Severity Score for the enUmeration of Mortality and Morbidity (***P-POSSUM***)

## Discussion

The aim of this scoping review was to provide a comprehensive overview of all risk factors used in the published literature on online calculators, equations, nomograms, and artificial intelligence models predicting POPF and other postoperative complications following PD. Furthermore, to summarize the externally validated risk models that incorporate POPF and other complications. Since the morbidity rate following PD is high, prediction of postoperative complication risk is crucial for patient counselling, informed consent, and adjustments in surgical strategy [4, 14]. Prediction of complication aids the surgeon to inform patients about the possible complications and assists them in the shared decision process [150]. In addition, identification of potential complications helps the surgeon to select the most appropriate surgical technique for each individual patient, for example, the decision to perform total pancreatoduodenectomy instead of pancreatojejunostomy or isolated roux-limb reconstruction techniques [151, 152]. 

Some risk factors can be assessed preoperatively, whereas other risk factors can only be estimated intraoperatively or postoperatively. Some studies use preoperative risk factors to calculate complication risk such as BMI, age, gender, and ASA score [8, 14, 45]. In our study, 39 of the 90 included studies (43.3%) used solely preoperative risk factors. Amongst the commonly reported intraoperative risk factors are soft pancreatic texture, narrow main pancreatic duct diameter and intraoperative blood loss [2, 6, 13]. Including intraoperative and postoperative factors in risk scores renders them unsuitable to predict risk of complications during preoperative patient counselling [153]. Pathological findings as a risk factor were used as a preoperative predictor depending on diagnostic biopsy findings in some studies or as a postoperative predictor in other studies depending on the definitive pathological assessment of the resected specimen. Furthermore, many of the risk scores use several variables and some of them are not designed specifically for PD rather than other surgeries as well [84, 150]. 

The most reported independent risk factors for POPF that were used to calculate the fistula risk were the main pancreatic duct diameter followed by pancreatic texture. This was probably due to the fact that the main pancreatic duct diameter can be assessed reliably using CT/MRI prior to surgery, whereas pancreatic texture is usually evaluated subjectively by the surgeon during surgery [150]. Although most studies claim that the pancreatic texture could only be evaluated intraoperatively, some studies have illustrated that this could also be achieved preoperatively using a dynamic MRI or CT-scan [40, 154]. Some authors consider soft pancreatic texture as the most predictive risk factor for POPF risk [4, 155]. Recently, the International Study Group of Pancreatic Surgery (ISGPS) has made a consistent classification regarding the risk factors of POPF after PD [156]. This classification involves four categories using a combination of main pancreatic duct diameter and pancreatic textures which are the most used risk factors in risk stratification. The ISGPS suggested using this classification for future reporting of POPF risk factors [156]. Furthermore, one study has investigated the risk factors associated with the development of NAFLD. The incidence of NAFLD after pancreaticoduodenectomy (PD) was 37% of which in 10% of the cases the diagnosis was confirmed with a biopsy. The authors stated that the reason is not yet completely understood. A previous study by Tanaka et al. suggested that there might be an association between pancreatic exocrine insufficiency and NAFLD due to pancreatic maldigestion [87, 157].

Interestingly, chronic pancreatitis and pancreatic cancer patients develop less POPF than those with other diagnoses. This is due to the presence of pancreatic fibrosis and a wide main pancreatic duct [13]. Pancreatic fibrosis is associated with a firm pancreatic texture, which acts as a protective factor against POPF [4]. Patients with pancreatic cancer are also more likely to have hard pancreatic texture in reaction to the obstructive tumor. A hard texture fibrotic pancreas releases fewer enzymes compared to a soft texture pancreas [4]. Therefore, a soft texture pancreas could lead to more proteolytic enzymes being secreted and subsequent leakage may occur, which enhances pancreatic fistula formation [13, 158]. Patients with duodenal cancer have the highest bile leak rate after PD and this is perhaps due to the narrow bile duct [159]. In fact, it appears to be less difficult to perform pancreatic anastomosis in patients having a hard pancreas with a wide main pancreatic duct, than vice versa [9]. It is known that soft pancreatic texture and narrow pancreatic duct diameter increase the risk of POPF. This might be due to failure of anastomosis as a consequence of inadequate suture holding ability in a soft pancreas or suture compression which may cause ischemia and lead to failure of anastomosis. Moreover, narrow pancreatic duct diameter increases the risk of POPF due to anastomosis challenge [151]. In a study regarding the impact of neoadjuvant chemoradiation and radiotherapy on the incidence of POPF, van Dongen et al. demonstrated that there was a reduction of POPF rate after chemoradiation or radiotherapy. This could be due to the fact that pancreatic texture becomes more firm as a result of fibrosis due to the radiation [160]. Several studies have been performed to investigate which anastomotic reconstruction technique is the best for preventing POPF. However, no anastomotic technique was found to be superior to one another [9]. Finally, the incidence of POPF is higher in male patients, which could be explained by differences in fat distribution between males and females, as males have more visceral fat [33]. Risk stratification of POPF can help in the decision-making regarding drain management. There are numerous studies conducted to investigate the association between drain amylase level and POPF. Drain placement and removal are still a subject of debate [161]. A study by Chang J et al. suggested that drain amylase level of ≥ 720 U/L in the postoperative day one is a good cutoff point for POPF [162]. Early drain removal might be useful in reduction of complications and infection [161]. There have been suggestions of corticosteroid use to reduce postoperative complications following PD. A randomised controlled trail (RCT) of 155 patients who received hydrocortisone during the operation showed an overall reduction in complications [163]. However, a more recent double-blind, multicenter RCT involving 428 patients who received dexamethasone during the operation showed no significant reduction in the complication rate [164]. 

Of the included 70 studies about POPF and other complications, only 18 studies (26%) were externally validated in an independent cohort of patients. This percentage was lower for the risk models using artificial intelligence, which was 20%. The focus of most research in this field was to develop new risk models rather than model validation and implementation in clinical practice. It is important to validate risk prediction models externally and measure their performance before implementation in clinical practice [165]. Currently the most cited and validated risk score is the original fistula risk score (o-FRS) by Callery et al. [31] This score is based on four preoperative and intraoperative risk factors namely main pancreatic duct diameter, pancreatic texture, estimated perioperative blood loss and definitive pathology [31].

This score uses no patient related risk factor, imaging, or laboratory findings. The a-FRS uses BMI, pancreatic duct diameter measured using preoperative CT scan and pancreatic texture as risk factors [34]. The ua-FRS uses BMI and age as patient related factors besides pancreatic duct diameter and pancreatic texture as pancreas related factors evaluated during surgery [33]. For clinical implementation, we recommend a risk calculator that uses a few risk factors, that contains related preoperative factors from the patient, pancreas, laboratory, and imaging. It should be available online and externally validated in a large cohort or multicentre. Finally, based on this scoping review study, we strongly recommend future research in this field to validate existing risk scores that have not been validated yet. External validation of risk models provides relevant insights in model performance (e.g. model robustness and generalizability) when applied to different group of patients, which is deemed necessary for responsible implementation in clinical practice.

### Strengths and limitations

With this scoping review, we aimed to offer a complete summary of the risk factors used in the published literature on risk calculators, equations, nomograms, and artificial intelligence prediction models for POPF and other severe complications following PD. The results of this study could help surgeons to acquire a better understanding of the possible risk factors that could influence the occurrence of POPF and other complications.

We acknowledge that the current study has several limitations. First, only the studies published in the English language were included. This might limit the generalizability of the study. Second, this study was restricted to pancreatoduodenectomy, and other types of resections were excluded. Third, due to the nature of the study, the outcomes may lack depth, as the review focuses on a broad range of topics.

## Conclusion

The main pancreatic duct (MPD) diameter was the most used factor when stratifying the risk of POPF followed by pancreatic texture, whereas age and ASA score were the most used factor when stratifying the risk of other complications. BMI was a commonly used risk factor for both. A better understanding of the used risk factors for POPF and other complications after PD could lead to refinement in risk stratification, improvement of patient counselling and informed consent, and adaptations in surgical strategy. This study revealed that only 18 of POPF and other complications studies (26%) were externally validated in an independent group of patients. This percentage was even lower for the risk models using artificial intelligence, which was (20%), indicating that most research in this field focuses on developing new risk models rather than validation and clinical implementation. We suggest future research to focus on external validation of existing risk models, providing relevant insights in model performance as a solid basis for responsible clinical implementation and decision-making.

## Figures and Tables

**Fig. 5:**
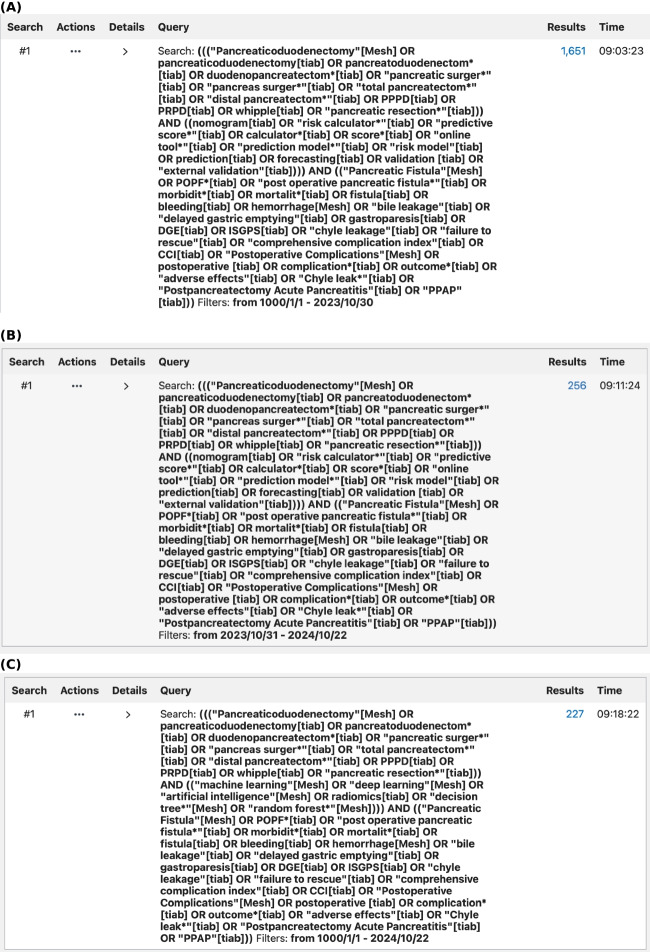
Search strategy used in PubMed. **(A)**: Initial search until October 30,2023. **(B)**: Search after update until October 22, 2024. **(C)** Search strategy used to identify artificial intelligence risk models until October 22, 2024

**Table 6.**
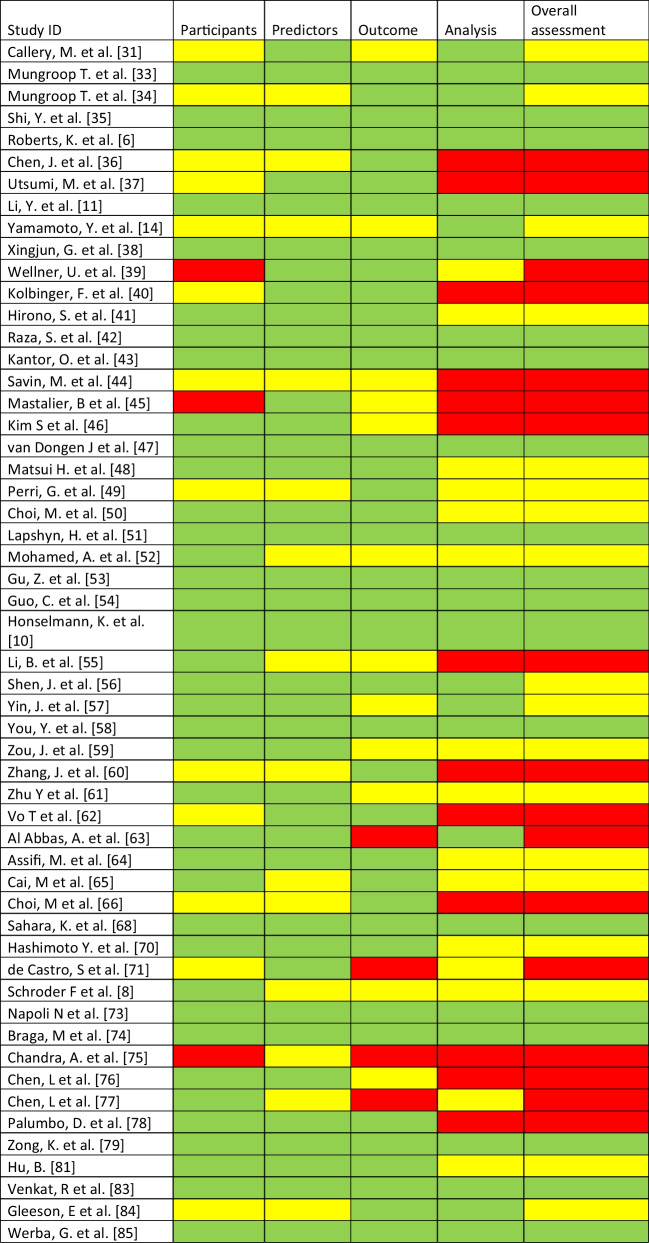

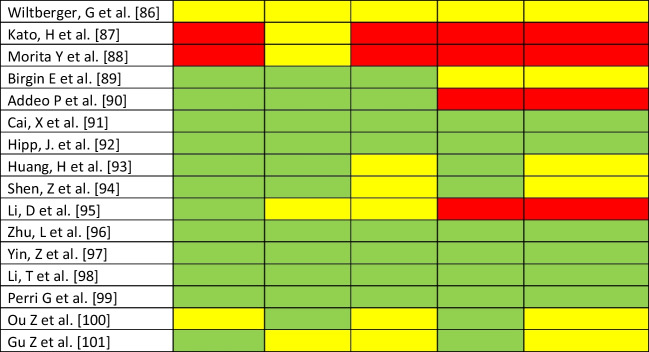
Risk of bias assessment using the PROBAST (Prediction model risk of bias assessment tool)

## Data Availability

No datasets were generated or analysed during the current study.

## Appendix 1

5

## Appendix 2

5

**Table 5:** Preferred reporting items for systematic reviews and meta-analyses extension for scoping reviews (PRISMA-ScR) checklist

SECTION	ITEM	PRISMA-ScR CHECKLIST ITEM	REPORTED ON PAGE #
TITLE			
Title	1	Identify the report as a scoping review.	1
ABSTRACT			
Structured summary	2	Provide a structured summary that includes (as applicable): background, objectives, eligibility criteria, sources of evidence, charting methods, results, and conclusions that relate to the review questions and objectives.	1
INTRODUCTION			
Rationale	3	Describe the rationale for the review in the context of what is already known. Explain why the review questions/objectives lend themselves to a scoping review approach.	1–2
Objectives	4	Provide an explicit statement of the questions and objectives being addressed with reference to their key elements (e.g., population or participants, concepts, and context) or other relevant key elements used to conceptualize the review questions and/or objectives.	2
METHODS			
Protocol and registration	5	Indicate whether a review protocol exists; state if and where it can be accessed (e.g., a Web address); and if available, provide registration information, including the registration number.	N/A
Eligibility criteria	6	Specify characteristics of the sources of evidence used as eligibility criteria (e.g., years considered, language, and publication status), and provide a rationale.	2
Information sources*	7	Describe all information sources in the search (e.g., databases with dates of coverage and contact with authors to identify additional sources), as well as the date the most recent search was executed.	2
Search	8	Present the full electronic search strategy for at least 1 database, including any limits used, such that it could be repeated.	2 + appendix 1
Selection of sources of evidence†	9	State the process for selecting sources of evidence (i.e., screening and eligibility) included in the scoping review.	2
Data charting process‡	10	Describe the methods of charting data from the included sources of evidence (e.g., calibrated forms or forms that have been tested by the team before their use, and whether data charting was done independently or in duplicate) and any processes for obtaining and confirming data from investigators.	N/A
Data items	11	List and define all variables for which data were sought and any assumptions and simplifications made.	2–4
Critical appraisal of individual sources of evidence§	12	If done, provide a rationale for conducting a critical appraisal of included sources of evidence; describe the methods used and how this information was used in any data synthesis (if appropriate).	4
Synthesis of results	13	Describe the methods of handling and summarizing the data that were charted.	2–3
RESULTS			
Selection of sources of evidence	14	Give numbers of sources of evidence screened, assessed for eligibility, and included in the review, with reasons for exclusions at each stage, ideally using a flow diagram.	3–4
Characteristics of sources of evidence	15	For each source of evidence, present characteristics for which data were charted and provide the citations.	4
Critical appraisal within sources of evidence	16	If done, present data on critical appraisal of included sources of evidence (see item 12).	Appendix 3
Results of individual sources of evidence	17	For each included source of evidence, present the relevant data that were charted that relate to the review questions and objectives.	4–13
Synthesis of results	18	Summarize and/or present the charting results as they relate to the review questions and objectives.	4–13
DISCUSSION			
Summary of evidence	19	Summarize the main results (including an overview of concepts, themes, and types of evidence available), link to the review questions and objectives, and consider the relevance to key groups.	13–14
Limitations	20	Discuss the limitations of the scoping review process.	20
Conclusions	21	Provide a general interpretation of the results with respect to the review questions and objectives, as well as potential implications and/or next steps.	20–21
FUNDING			
Funding	22	Describe sources of funding for the included sources of evidence, as well as sources of funding for the scoping review. Describe the role of the funders of the scoping review.	25

*JBI*, Joanna Briggs Institute; *PRISMA-ScR*, Preferred Reporting Items for Systematic reviews and Meta-Analyses extension for Scoping Reviews

*Where *sources of evidence* (see second footnote) are compiled from, such as bibliographic databases, social media platforms, and Web sites

†A more inclusive/heterogeneous term used to account for the different types of evidence or data sources (e.g., quantitative and/or qualitative research, expert opinion, and policy documents) that may be eligible in a scoping review as opposed to only studies. This is not to be confused with *information sources* (see first footnote).

‡The frameworks by Arksey and O’Malley (6) and Levac and colleagues (7) and the JBI guidance (4, 5) refer to the process of data extraction in a scoping review as data charting

§The process of systematically examining research evidence to assess its validity, results, and relevance before using it to inform a decision. This term is used for items 12 and 19 instead of "risk of bias" (which is more applicable to systematic reviews of interventions) to include and acknowledge the various sources of evidence that may be used in a scoping review (e.g., quantitative and/or qualitative research, expert opinion, and policy document)

## Appendix 3

6.
